# Examining the Individual Response to a Low-Sodium Diet in Patients with Hypertension: Protocol for a Pilot Randomized Controlled Trial

**DOI:** 10.2196/39058

**Published:** 2023-02-13

**Authors:** Jisook Ko, Jing Wang, Misook L Chung, Kumar Sharma

**Affiliations:** 1 School of Nursing University of Texas Health San Antonio San Antonio, TX United States; 2 College of Nursing Florida State University Tallahassee, FL United States; 3 College of Nursing University of Kentucky Lexington, KY United States; 4 Department of Medicine University of Texas Health San Antonio San Antonio, TX United States

**Keywords:** adherence to low-sodium diet intervention, adherence, metabolite profile, salt sensitivity, digital self-monitoring, hypertension, salt, blood pressure, sodium, diet, metabolite, nutrition, metabolomics, precision medicine, personalized, personalization, RCT, randomized controlled trial, genetics, genomics, chronic disease, chronic condition, urinary, dietary, dietary sodium, hypertensive patient, cardiovascular disease

## Abstract

**Background:**

Excessive dietary sodium intake is an independent risk factor for hypertension and cardiovascular disease (CVD). Despite the large body of evidence concerning the effects of dietary interventions on blood pressure (BP) and CVD outcomes, trials have often reported low adherence to decreased sodium intake, likely due in part to heterogeneous BP responses. To address the challenges, recent clinical findings suggested a precise and personalized dietary approach that seeks to deliver more preventive and practical dietary advice than the “one-size-fits-all” guidelines and weighs the personal risk of developing specific diseases.

**Objective:**

The purpose of this pilot randomized controlled trial was to test the feasibility and preliminary efficacy of integrating the use of mobile technology and metabolomics with a low-sodium diet intervention in patients with hypertension to develop personalized low-sodium diet programs. Additionally, the study will examine the associations of urine metabolites with urinary sodium levels and BP control based on the hypothesis that targeted urine metabolites. In this report, we describe the design and protocol of the pilot trial.

**Methods:**

A total of 40 patients with hypertension will be randomly assigned to either a 8-week low-sodium diet group (n=20) or a standard care group (n=20). Each week, intervention participants went through individual sessions with an interventionist via videoconferencing to discuss low-sodium diet regimens, patients’ food choices, and BP tracks on mobile apps. The control group followed their usual care for hypertension management. All participants in both groups monitored diet and BP using mobile apps for 8 weeks. A 24-hour urinary sodium excretion for the estimation of dietary sodium intake, systolic, and diastolic BPs were measured at the baseline and at 8 weeks. The primary outcomes of this study include the feasibility of conducting a randomized controlled trial (RCT) by reporting recruitment, retention, and completion statistics. The preliminary effects of intervention will be tested by a generalized estimating equation model.

**Results:**

This pilot RCT study was approved by the institutional review board at the University of Texas Health San Antonio in January 2021. The first participant was enrolled in April 2021, and currently, 26 participants were enrolled. All data collection is expected to conclude by March 2023, with data analysis and study results ready for reporting by December 2023. Findings from this pilot RCT will further guide the team in planning a future large-scale study.

**Conclusions:**

The findings of this proposed study will establish a comprehensive knowledge base for future research and development of personalized dietary interventions to promote adherence to dietary strategies and self-management of chronic disease using the Precision Health approach for millions of Americans who are struggling with uncontrolled hypertension.

**Trial Registration:**

ClinicalTrials.gov NCT04764253; https://clinicaltrials.gov/ct2/show/NCT04764253

**International Registered Report Identifier (IRRID):**

DERR1-10.2196/39058

## Introduction

Excessive dietary sodium intake is an independent risk factor for hypertension and cardiovascular disease (CVD). CVD is the leading cause of death in the United States. Risk factors for CVD include obesity, diabetes, and hypertension. Nutritional practices are a critical component in the prevention of CVD [1,2]. It is well documented that excess sodium intake can lead to hypertension, the primary risk factor for CVD [3]. Indeed, among all risk factors for CVD, elevated intake of sodium alone was ranked the 11th most important [4]. The scale of this problem resulted in calls for dietary sodium reduction to be considered a public health priority. Sodium intake is included in the top 5 priority actions for chronic disease control [5]. Despite this effort, daily dietary sodium intake among Americans chronically exceeds recommendations (2300 mg per day).

Numerous clinical trials demonstrated that a low-sodium diet reduces the risk of cardiovascular outcomes, including the incidence of hypertension, stroke, and cardiovascular morbidities [6-8]. Most recently, a systematic review and meta-analyses demonstrated that lowering sodium intake significantly reduced resting systolic blood pressure (BP) by 3.39 mm Hg and resting diastolic BP by 1.54 mm Hg [9]. When sodium intake was < 2 g/day versus ≥ 2 g/day, systolic BP was reduced by 3.47 mm Hg and diastolic BP by 1.81 mm Hg [9]. These changes seem small but are significant.

Prior dietary interventions successfully reduced sodium intake, but adherence to these dietary strategies is difficult to maintain [10,11]. This issue is likely due in part to the heterogeneity of BP responses to alterations in dietary sodium intake. In addition to examining group average BP responses to sodium intake, BPs of some—but not all—individuals fall with reduction in sodium intake, leading to the concept of intraindividual variability in BP response to a low-sodium diet [11-14]. Because there is considerable intraindividual random daily variation in BP without a change in sodium intake, both individual and intraindividual random variations in BP must be considered [15]. This proposed pilot study aims to address these challenges and investigate whether a low-sodium diet would induce changes in metabolic profiling and reflect intraindividual variability in response to a low-sodium diet among patients with hypertension, and also measure the association with reduced BP.

In recent years, precision health is often described in relationship to precision medicine, an emerging approach for disease treatment and prevention that accounts for intraindividual variability in genes, environment, and lifestyle [16]. Precision or personalized nutrition focuses on ways to deliver tailored dietary recommendations to promote and maintain health and prevent disease [17]. However, this field is in its infancy and currently has limited clinical applicability to CVD.

Metabolomics can measure the full profile of small-molecule metabolites in biofluids and provide a comprehensive picture of a person’s overall dietary intake [18]. Diet encompasses a complex set of intercorrelated exposures, and self-reported tools are prone to random and systematic errors [18]. Although several biomarkers that measure nutrient intake have been identified, objective measurement of the overall dietary pattern remains elusive. Metabolite profiling accounts for intrinsic variability in metabolism by measuring downstream components or metabolic products of foods and, therefore, may more accurately measure true exposure than the traditional methods that measure individual food intake [18]. Although a few studies [19,20] identified metabolites associated with sodium intake, little research addresses metabolic phenotyping in relation to a low-sodium diet. This project aims to examine the variance in metabolite profiling caused by a low-sodium diet while controlling for covariates such as age and gender and provide the preliminary findings that estimate the variance of a modifiable factor (a low-sodium diet) in BP control.

Salt sensitivity (SS) as a preceding factor for individual BP response to a low-sodium diet can be tested with a genetic approach. SS is influenced by many factors, including genetics, age, gender, race or ethnicity, BMI, and diet [20,21]. The most robust method for assessing SS is through modifying dietary sodium intake using a crossover study design where a 5-7–day intervention period is given for a normal, low, and high dietary sodium intake level [22,23]. When a change in BP is not observed despite changes in sodium chloride intake, the individual is classified as salt resistant. Validated rapid tests or diagnostic markers to identify SS of BP in clinical practice were not available until now. In the most recent published study, which used data from the Genetic Epidemiology Network of Salt Sensitivity, GNAI2 single-nucleotide polymorphisms (SNPs) were positively associated with SS independently of subject sex or age [24]. Examining SS in BP using a genetic approach will enhance the identification of individuals at high risk for developing hypertension and would also benefit from a low-sodium diet.

Mobile apps that monitor food intake have the potential to improve eating habits and promote healthy diets. Although most popular apps are intended to monitor calorie intake to promote weight loss, they could also be used to monitor specific nutrient concerns such as dietary levels of sodium or monitor adherence to therapeutic dietary plans. One pilot study using a smartphone app showed that the change in the predicted 24-hour sodium excretion significantly differed between groups using an app versus a group recording intake in a journal (mean change −838, SD 1093 and 236, SD 1333 mg, respectively) [25]. Thus, further research is needed to explore whether mobile apps facilitate the real-time assessment of dietary intake as a means to improve self-managing dietary behavior using more precise monitoring [26].In conclusion, despite the benefits of low-sodium diet programs for reducing high BP and improving CVD outcomes in patients with hypertension, much of the existing empirical support for low-sodium diet programs are based on the evidence from a one-size-fits-all approach and has not accounted for individual responses to diet modification. Using a precision nutrition approach, we can address these gaps by providing more personalized interventions that may enhance adherence to a low-sodium diet and improve sustainability for individuals with hypertension. However, research developing and implementing a personalized low-sodium diet for patients with hypertension is limited, and more foundational evidence is needed.

## Methods

### Study Design and Overview

We are currently conducting a randomized controlled trial to test the feasibility of integrating the use of mobile technology and metabolomics with a low-sodium diet program compared to standard care (control). We will randomly assign 20 participants with hypertension to each group (N=40) to the intervention or the control group. The intervention is 8 weeks long and we will measure adherence and BP outcomes as the primary end points.

### Ethical Considerations

This study is approved by the University of Texas (UT) Health Science Center at San Antonio’s Institutional Review Board (#HSC20200054H). All databases containing personal data will be encrypted to minimize the risk of a breach. In addition, all the servers where data are stored will be encrypted and require a one-time password to be made available for analysis. Participants who completed the baseline visit and follow-up visit received US $60 of ClinCards per institutional policy as compensation for their time.

### Specific Aims and Hypotheses

The aims of this clinical trial are to (1) determine the feasibility and preliminary efficacy of delivering a low-sodium diet intervention focused on changes in metabolomic profiling and reduction of urinary sodium level and BP in patients with hypertension; (2) examine the associations of urine metabolites with urinary sodium levels and BP control based on the hypothesis that targeted urine metabolites will have significant associations with urinary sodium level and differences in systolic BP (SBP) and diastolic BP (DBP).

### Study Setting

The UT Health San Antonio School of Nursing will serve as the coordinating center for this study. Eligible participants will be recruited from primary care centers at UT health physicians. Data will be collected in the UT Health San Antonio School of Nursing.

### Sample

#### Sample Size Justification and Power

This is a pilot study and will be inherently underpowered; therefore, quantitative outcomes will be interpreted only as feasibility and pilot data. A sample size of 40 subjects will provide an adequate sample size [27] to calculate meaningful CIs for our estimates of feasibility and preliminary effects. With this sample size, we expect to have sufficient diversity of input on the intervention components to plan refinements for a larger intervention trial.

#### Inclusion and Exclusion Criteria

To examine the underlying mechanism of intraindividual response to a low-sodium diet, a randomized, controlled study will be conducted in a sample of 40 patients with hypertension aged >40 years. Children are thought to have minimal levels of high BP; therefore, we will only recruit adults. Participants will be asked to own their own smartphone for using mobile apps during the study. Our exclusion criteria are clinic BP of <140 mm Hg; estimated glomerular filtration rate of <15 mL/min/1.73 m^2^ or on dialysis; history of organ transplantation; cardiovascular event, procedure or hospitalization for unstable angina/chronic heart failure within last 6 months; life expectancy of <1 year or a cancer diagnosis and treatment within the past 1 year that, in the judgment of clinical study staff, would compromise participant’s ability to comply with the protocol and complete the trial; active alcohol or substance abuse within the last 12 months; residence in a nursing home; diagnosis of dementia, treatment with medications for dementia; and unwilling or unable to participate in either of the interventions.

### Measures

After obtaining informed consent, we will begin collecting baseline data and schedule the 8-week study visit. The summary of variables, measures, and time of evaluation is presented in Table 1.

**Table 1:** Factors, variables, measures, and time data collected.

Factors and variables		Measures	Time data collected
**Individual characteristics**			
	Age	Years	Baseline
	Sex	Male and female	Baseline
	BMI	Weight and height	Baseline and 8 weeks
**Physiological factors**			
	Metabolite profile	24-hour urinary sample	Baseline and 8 weeks
	Salt sensitivity gene	Single-nucleotide polymorphism genotyping	Baseline
**Feasibility measures**			
	Retention rate	Number of subjects that came to the follow-up visit	Over 8 weeks
	Attendance at education sessions	Number of education sessions attended on the internet	Over 8 weeks
	Adherence to dietary sodium intake monitoring	Logging a meal in the app/manual	Over 8 weeks
	Adherence to in-home blood pressure monitoring	Logging BP using BP monitor device	Over 8 weeks
**Outcomes**			
	Blood pressure	WelchAllyn Vital Signs Monitor	Baseline and 8 weeks
	Sodium intake	24-hour urinary sodium excretion	Baseline and 8 weeks

#### Individual Characteristics

Age and sex will be self-reported. BMI will be calculated using height and weight measurements and obtained with an Accu-Hite wall mounted stadiometer and a Withings electronic scale, respectively.

#### Physiological Factors

Metabolite profile will be measured by using targeted capillary electrophoresis/mass spectrometry (CE/MS) for 48 amino metabolites from 24-hour urine samples collected at baseline and at 8 weeks.

The SS gene will be screened using targeted genotyping. The targeted gene under investigation will be GNAI2 SNPs, which are positively associated with SS independently of sex and age. The primary SNPs investigated will be rs10510755, rs9852677, rs2282751, rs4547694, and rs2298952 identified from the HapMap and 1000Genomes projects to capture 100% of the genetic variation in GNAI2 using the Affymetrix Genome Wide-Human SNP Array AFFY_6.0.

#### Feasibility Measures

Four feasibility measures will be used to help elucidate our understanding of using a low-sodium diet intervention with mobile technology and metabolomics to measure intervention compliance intervention and protocol adherence.

Retention rate is the percentage of subjects who attended the 8 weeks post intervention. Attendance to education sessions will be measured by the number of 8-week education sessions attended within intervention group.

We will measure adherence to dietary sodium intake monitoring with the Fitbit companion mobile dietary app. The Fitbit app is intended as an intervention tool to enhance self-monitoring and promote adherence, not as a reliable assessment tool for dietary sodium intake (the 24-hour urinary samples will be used for this purpose). Each subject will register and assigned a unique ID in the platform developed by the Center on Smart and Connected Health Technologies at UT Health San Antonio. Each Fitbit account will be linked to his or her unique ID through this platform. Subjects will be instructed to record food intake in real time. Adherence to sodium intake monitoring is measured by calculating the percentage of days a participant logs at least 1000 kcal his or her meals in the dietary app.

Adherence to in-home BP monitoring is the percentage of days a subject uses the BP monitoring device (Omron 7 Series Upper Arm BP Monitor) to log BP. It has a range of 0 to 299 mm Hg for BP and 40 to 180 beats per minute for heart rate. The cuff is inflated with an electric pump and deflated with a pressure release valve. SBP, DBP, and heart rate are displayed on the LCD screen after each measurement. The device can also display a symbol on the screen, indicating an irregular heartbeat detected during measurement of SBP and DBP. Each subject will register for an Omron app account, which will be linked to their Connected Health platform unique ID. We will provide instructions on how to measure BP while in a sitting position and transfer the BP data to Omron mobile app using the BP monitoring device.

#### Outcomes

BP will be measured with the Vital Signs Monitor 300 Series model (WelchAllyn) at baseline and 8-week follow-up appointments. The midsection circumference of the dominant upper arm will be measured with a tape measure [28], and the proper sized cuff will be selected accordingly. SBP and DBP will be measured in the dominant arm twice with a 2-minute rest period between measures [29]; the 2 measures will be averaged. Sodium intake will be measured using a 24-hour urinary sodium excretion (mg excreted/day) test collected at baseline and at 8 weeks post intervention.

### Procedures

#### Recruitment

Recruitment began in April 2021. After obtaining approval from the institutional review board, subjects will be informed about the study via (1) UT Health San Antonio internal IT systems (ie, email and clinical trials website), (2) using Epic system to identify potentially eligible patients and send MyChart invitations, (3) study brochures posted at the primary care centers, and (4) word of mouth. For interested potential subjects, the principal investigator arranged a convenient time for consent process and screening for eligibility.

#### Informed Consent, Screening, and Randomization

After explaining the study’s purpose, procedures, and potential benefits and risks in greater detail, we will obtain informed consent and screen BP (Table 2). For those meeting all criteria, we will arrange a convenient date and time for data collection in the Biobehavioral Research Laboratory. To prevent subject attrition, we will send appointment reminders 1 day prior to the scheduled visit. We will randomly assign the subjects into 1 of 2 groups using block randomization to balance sample sizes across groups [30].

**Table 2:** Procedures.

Variables	Screening (30 minutes)	Pretest (60 minutes)	Intervention (8 weeks)	Posttest (30 minutes)
Information session	N/A^a^	A low-sodium diet intervention Using wireless, wearable, and a mobile app 24-hour urine collection	N/A	N/A
Self-report	Age Informed consent	N/A	Dietary sodium intake In-home blood pressure monitoring	N/A
Noninvasive measure	Blood pressure	Height and weight Blood pressure 24-hour urinary excretion	N/A	Height and weight Blood pressure 24-hour urinary excretion
Blood collection	N/A	Salt sensitivity gene	N/A	N/A

*^a^*N/A: not applicable.

#### Baseline Data Collection

After measuring height and weight, BP will be measured twice with a 2-minute rest period between the measurements. Next, we will draw 4.0 mL of blood by venipuncture. The research team will provide a low-sodium diet intervention tutorial and hold an information session to explain the mobile app, Fitbit wristband, and the in-home BP monitoring system. Subjects will be instructed to create user accounts for each device and receive hands-on training for dietary sodium intake and BP level self-monitoring using the Fitbit and wireless device. We also will provide participants with a designated helpline number for technical support. We will issue a designated container and instructions for 24-hour urinary collection. Subjects will be asked to note the start time in a written log and discard the first urine, then collect all urine over the next 24 hours, noting the time and amount excreted for each collection in the written log. Stored urine will not require refrigeration. In total, 4.0 mL of peripheral blood will be drawn into an anticoagulant tube during baseline visit. The anticoagulant tube will be centrifuged at 4000 rpm at 4 °C for 10 minutes. The Illumina Infinium system will be used for high-throughput genotyping of SNPs using microarrays. The 24-hour urine sample will be analyzed using the targeted capillary electrophoresis/mass spectrometry method for 48 amino metabolites.

#### Group Conditions

The intervention consists of 8 educational sessions (45 minutes each) delivered via a videoconferencing program and accessed through personal computer or smartphone at participants’ preferred time. In the control condition, 20 subjects will receive their routine medical and nursing care for hypertension and take medications as prescribed. In both groups, over the 8-week intervention, participants will be allowed in digital self-monitoring using Fitbit, its companion app, and wireless BP monitor (Table 3).

**Table 3:** Major content of intervention sessions.

Session	Content
1. Getting started and blood pressure overview	Introduction to the program Education about blood pressure and hypertension Discussion of symptoms and complications for uncontrolled hypertension
2. A low-sodium diet and digital self-monitoring	Education about a low-sodium diet in hypertension Discussion of the importance of digital self-monitoring of diet and taking blood pressure at home
3. Gradual dietary sodium reduction	Education about the rationale for gradual adaptation strategy, physical gradual adaptation process in taste buds, and psychological adaptation process Setting up weekly goals in gradual adaptation
4. Strategies to overcome barriers to adhere to a low-sodium diet	Education about individual’s barriers to adhere to a low-sodium diet including cooking, eating out at restaurants, grocery shopping, salt substitutes or seasonings, fast food choice, and menu development
5. Food labels and sodium	Education about reading food labels and nutrition facts Facts about salt and sodium, and tips for how to lead a low-sodium life Revisiting the gradual sodium reduction goal
6. Serving sizes and portion control	Discussion about serving sizes, portion control, and the plate method Reviewing the gradual sodium reduction goal
7. Meal planning, shopping, and eating out	Discussion of planning for meals and tips for shopping and eating out Checking in on goal progress
8. Planning for lasting change	Review of overall program content Discussion of what worked with a low-sodium diet adherence Planning for lasting change activity

#### Follow-up Data Collection

After completion of the intervention, the PI will ask all participants to measure BMI, BP, and collect a 24-hour urine sample to measure sodium excretion in the same manner as the baseline.

### Data Analytic Plan

Data analysis will be performed with SAS (version 9.4; SAS Institute) and R (version 4.1+; RStudio, Inc). Baseline demographic information will be summarized using descriptive statistics. Continuous variables will be summarized with means and SDs. Categorical variables will be summarized with counts, proportions, and medians (IQR). The distribution of all variables will be examined before any analysis, using appropriate statistical tests like the Shapiro test for normality checking. If the assumption of normality is not met, equivalent nonparametric approaches or data transformation will be used; for instance, log transformation can be conducted to the metabolomics profile before standardized to unit variance and zero mean. The multiple testing problem will be accounted for using Bonferroni correction or the Benjamini-Hochberg procedure: for example, the false discovery rate will be controlled when testing multiple urinary metabolites. All testing will be 2-sided, and a P value of .05 will be used as the family-wise significance level. If needed, statistical analysis will also be conducted after controlling for age, sex, and BMI.

#### Aim 1

Using both pre- and posttest data, we will assess feasibility by measuring (1) the percentage of subjects present at the 8-week follow-up, (2) the number of education sessions attended on the internet (intervention group), (3) the percentage of days subjects logged meals using a mobile dietary app; and (4) the percentage of days subjects logged in-home BP measurements with the BP monitoring device. We will test the preliminary effect of a low-sodium diet on the outcome variables using a generalized estimating equation model to account for the repeated measures over 8 weeks, which includes fixed effects of time (baseline and 8 weeks) and study arm (intervention or control), an interaction term for time by arm, and other covariates such as age. The interaction term will be tested to assess whether the changes from baseline differ by groups.

#### Aim 2

Using pretest data, a random forest [31] using R (version 4.2.0) will be used to identify associations between 48 amino metabolites, BP phenotypes (including SBP or DBP), and 24-hour urinary excretion to predict the classification of study subjects. For urinary metabolites nominated by the random forest analysis, we will use generalized linear model to generate P values and false discovery rate to correct for testing multiple urinary metabolites using the Benjamini-Hochberg procedure.

## Results

To date, interventionist training is completed. The first participant was enrolled in April 2021, and currently, 26 participants were enrolled. All data collection is expected to conclude by March 2023, with data analysis and study results ready for reporting by December 2023. The findings from this pilot randomized controlled trial (RCT) will further guide the team in planning a future large-scale study.

## Discussion

### Findings and Implications

This report describes the protocol and design of a pilot RCT of a low-sodium diet intervention leveraging digital self-monitoring of daily diet and BP among patients with hypertension. We hypothesize that excessive sodium intake in individuals with hypertension can be modified with nutritional advice in addition to real-time diet and BP tracking and daily trends. Participants in the intervention group are anticipated to have a greater reduction in dietary sodium intake and BP than those in the control group. This study is the first attempt to develop a rigorously designed, low-sodium diet intervention to enhance metabolite response and dietary adherence for this vulnerable population. In addition, by examining metabolites to BP response and outcomes to a low-sodium diet, the findings of this study may establish a comprehensive knowledge base for future research and the development of precise and personalized nutrition regimens for high-risk individuals who are vulnerable to the negative effects of sodium on BP control based on genetic phenotypes and metabolite profiles. The findings of this proposed study will establish a comprehensive knowledge base for future research and development of personalized dietary interventions to promote adherence to dietary strategies and self-management of chronic disease using the Precision Health approach for millions of Americans who are struggling with uncontrolled hypertension.

### Strengths and Limitations

First, this study is a pilot randomized trial, the sample size is relatively small, and we are not adequately powered to detect statistical significances between groups. Second, study recruitment and retention will likely be the most challenging aspect, given the multiple web-based lessons and self-monitoring activities. We will monitor recruitment monthly, and if lagging, will expand recruitment clinics. We will also encourage enrollment by compensating participants with a US $30 gift card for completing each visit. We will use multiple strategies (eg, being flexible for scheduling appointments and sending motivational messages) to address and reduce dropout. By providing flexibility, we are successfully maintaining the enrollment of participants in the current pilot study over 8 weeks without dropout. Finally, potential confounding factors were not analyzed: randomization should address differences in other factors that may affect BP, such as physical activity, sleep, and comorbidity. These factors will be measured in a larger study.

Nonetheless, this study represents an initial step to examine the metabolite responses to integrating the use of mobile technology and metabolomics with a low-sodium diet intervention in patients with hypertension to develop personalized low-sodium diet programs. If the results are promising, this may serve as an important milestone in considering how to develop and implement personalized low-sodium diet interventions in millions of Americans who struggling with uncontrolled hypertension. The findings from this study will also serve as the foundation for a large full-scale RCT to test the program in a broad population.

## Abbreviations

BP: blood pressure
CE: capillary electrophoresis
CVD: cardiovascular disease
DBP: diastolic blood pressure
MS: mass spectrometry
RCT: randomized controlled trial
SBP: systolic blood pressure
SNP: single-nucleotide polymorphism
SS: salt sensitivity
UT: University of Texas

## Data Availability

The data sets generated or analyzed during this study are available from the corresponding author upon reasonable request.
